# Advances in cancer treatment: a new therapeutic target, Annexin A2

**DOI:** 10.7150/jca.55173

**Published:** 2021-04-24

**Authors:** Zinan Li, Lifeng Yu, Baohui Hu, Lianze Chen, Mingyi Jv, Lin Wang, Chenyi Zhou, Minjie Wei, Lin Zhao

**Affiliations:** 1 Department of Pharmacology, School of Pharmacy, China Medical University, No.77 Puhe Road, Shenyang North New Area, Shenyang City, 110122, Liaoning, China.; 2 Liaoning Engineering Technology Research Center, China Medical University, No.77 Puhe Road, Shenyang North New Area, Shenyang City, 110122, Liaoning, China.; 3 Liaoning Medical Diagnosis and Treatment Center, Liaoning Province, China.

**Keywords:** Annexin A2, cancer, target, cancer therapy

## Abstract

Annexin A2 (ANXA2) is a calcium regulated phospholipid-binding protein. It is expressed in some tumor cells, endothelial cells, macrophages, and mononuclear cells, affecting cell survival and mediating interactions between intercellular and extracellular microenvironment. Aberrant expression of ANXA2 can be used as a potential predictive factor, diagnostic biomarker and therapeutic target in cancer therapy. Investigators used various technologies to target ANXA2 in a preclinical model of human cancers and demonstrated encouraging results. In this review article, we discuss the diagnosis and prognosis latent capacity of ANXA2 in progressive cancers, focus on the exploration of restorative interventions targeting ANXA2 in cancer treatment. Further, we comment on a promising candidate therapy that is conceivable for clinical translation.

## Introduction

Annexin A2 (also called ANXA2, annexin II, p36), 39 kDa proteins (appearing as a 36 kDa protein by SDS-PAGE) from the calcium-dependent phospholipid-binding peripheral membrane proteins family 1, is characterized by the ability to bind and aggregate anionic phospholipid membranes 2. This calcium-dependent binding and aggregating ability makes up the foundation of its biological functions including vesicular transport, exocytosis, and endocytosis. Otherwise, ANXA2 participate in cell survival, proliferation, invasion and metastasis, thus acts as a regulator in tumor growth and progression, which support that ANXA2 is a proposing target in cancer treatment 3. The aberrant expression character of ANXA2 showed in a wide range of cancer cells turn it into emerging biomarker for cancers 4. In this review, we summarize the characteristics and roles of ANXA2 and existing therapeutic strategies targeting ANXA2, and propose a prospective compound targeting ANXA2.

## Characteristics of Annexin A2

There are two functional regions of ANXA2 underling its binding and aggregating activity: the N-terminal domain and the C-terminal core domain. The N-terminal domain contains the tissue plasminogen activator (tPA)- 5 and S100A10 (also called p11)-binding site, it also bears multiple phosphorylation sites such as Tyr23 and Ser25, which can be phosphorylated by Src kinase and protein kinase C 6, 7. The phosphorylated ANXA2 changes its intracellular localization and regulating actions 8-12. The core domain consists of four segments of internal and interannexin homology that are easily identified in a linear sequence alignment 13. It constitutes a highly α-helical disk with two principal sides, the convex and the concave 1, 14. The convex surface underlies membrane-binding ability in a calcium-dependent manner. The concave surface, meanwhile, plays a key role in membrane bridging and has the ability to anchor the C terminus of the N-terminal domain 15. The core domain includes the F-actin- 16, heparin- 17 and plasminogen-binding sites 18. It mediates the cycle of ANXA2 between cytosol and the cytosolic surface of cellular membranes through Ca^2+^-regulated way 19. The C-terminal core domain determines membrane-binding capability of ANXA2 20, while N-terminal domain underlies paralog-specific functions in ANXA2. It is pointed out that the N-terminal domain is necessary in targeting to endosomes *in vivo* 21. Tyr-23 phosphorylation in the N-terminal promotes the membrane-surface ANXA2 binding to endosomes 10. Reactive redox-sensitive cysteines residue (Cys-8) in the N-terminus can regulate cycles of oxidation and reduction 22. Extracellular ANXA2 bind to enzyme plasmin and oxidized during this reaction, oxidation form of ANXA2 subsequently reduced by the thioredoxin redox system. Reduced form of ANXA2 can protect tumor proteins from oxidation. Which means ANXA2 plays a key role in resisting oxidative stress and in tumorigenesis 23. Structural studies indicated that the flexibility of the N-terminal domain and C-terminal core domain is interrelated and oppositely regulated by Ser25Glu phosphomimicking mutation and Tyr23 phosphorylation. Ser25Glu mutation disrupts the connection of N-terminal domain and C-terminal core domain, while Tyr23 phosphorylation anchors the N-terminal domain to the C-terminal domain and inhibit the membrane-bridging function. This inhibition can be returned by S100A4 and S100A10 binding 24.

ANXA2 exists as monomeric and heterotetrameric (comprised of two ANXA2 and two S100A10 molecules) forms in cells. The monomer exists in the cell cytoplasm, nuclei and on early endosomes while the heterotetramer located on cell membranes 25. Nuclear ANXA2 can be the component of the primer recognition proteins regulating DNA polymerase α activity and DNA replication 26, 27. Nuclear ANXA2 is also a major nuclear DNA-binding protein and be associated with DNA synthesis, cell proliferation and cell cycle progression 28, 29. Otherwise, accumulation of ANXA2 in nuclear protects cells from DNA damage during oxidative stress 30. ANXA2 binds to specific mRNAs in cytoskeleton and perinuclear section. ANXA2 in the cytoplasm and on the surface of membranes can serve as mediators in membrane-related processes, including exocytosis 31, 32, endocytosis 25, 33, and membrane trafficking 34. A founding proved that ANXA2 involved in biogenesis of polycystic transport intermediates via regulating the budding process of early endosomes rather than membrane invagination 35. N-terminal domain, especially tyr-23 phosphorylation in the N-terminal, is necessary in this process 10, 21. The heterotetramer on the cell surface is regulated by intracellular calcium concentration. Change of intracellular calcium concentration can be provoked by heat induced stress of the cell. When using the small interfering RNAs (siRNA) which can specifically bind to S100A10 and subsequently reduce its expression, translocation of ANXA2 triggered by the heat was therefore significantly reduced. S100A10 plays an essential role in ANXA2 translocation 12. Calcium-dependent constructional change of ANXA2 exposes a hydrophobic amino acid, it combined with S100A10 and form the heterotetramer. The high affinity for phospholipids drives heterotetramer translocate from the cytoplasm to the extracellular plasma membrane 12, 36. The heterotetramer is a key plasminogen (PLG) receptor that transforms PLG into plasmin 37. Plasmin is an important regulator of ECM degradation, fibrin polymers lysis and furthermore migration, invasion and angiogenesis 38.

## Roles of Annexin A2 in cancer progression

**Figure 1:**
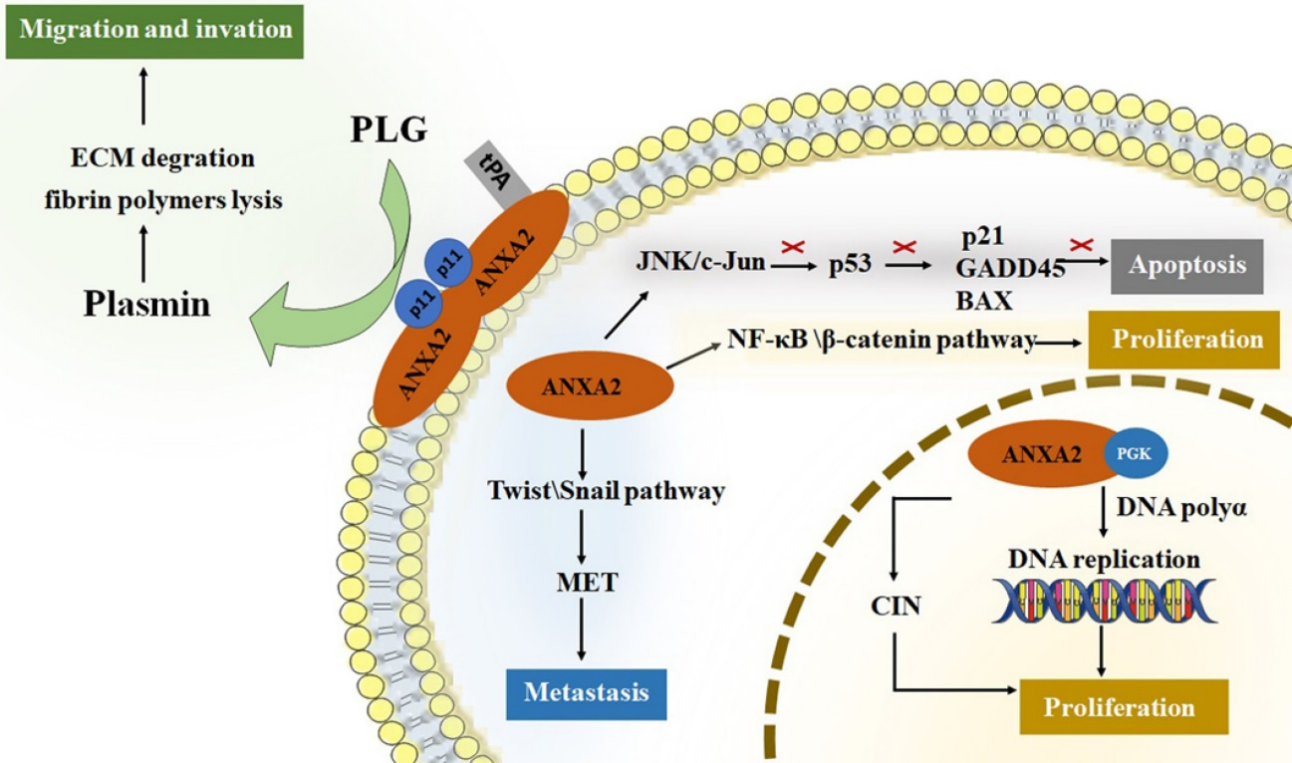
The roles of ANXA2 in cell development. ANXA2 represents essential promoter in cell proliferation, migration, invasion and metastasis, concurrently inhibit the apoptosis. tPA, tissue plasminogen activator; PGK, phosphoglycerate kinase; CIN, chromosome instability; PLG, plasminogen; ECM, extracellular matrix; EMT, epithelial-mesenchymal transition.

ANXA2 perform crucial roles in tumor cancer progression 3, 4, 39-45. For tumor development, promoting cell proliferation and inhibiting cell apoptosis are both required for survival and growth. Neoangiogenesis, meanwhile, is another fundamental biological process in tumor progression 46. It is well known that nutrition and oxygen are indispensable for tumor cells to survival and growth. So, in the early stage of tumor starvation, ANXA2 may support starving cells by inducing autophagy 47. Invasion and metastasis of tumor cells improve the aggressive potential of human cancer. Increased researchers have focused on roles of ANXA2 on proliferation, migration, invasion and metastasis of tumor cells. (*Figure 1*).

### Roles of Annexin A2 in cell proliferation

In nuclei, ANXA2 monomer combined with 3-phosphoglycerate kinase (PGK). This complex stimulates DNA polymerase alpha in the early S phase and initiates the DNA replication 27. In addition, nuclear ANXA2 disrupts coilin and causes its abnormally localized to centromeres, resulting in chromosome instability (CIN). CIN is a promotor of tumor growth 48-50. Study showed that cancer cells transfected with an antisense ANXA2 vector show poor capability of division and proliferation. Cellular DNA synthesis level in antisense transfected cells is significantly lower than that in sense transfected cells. Meanwhile, replication extracts made from antisense transfected cells have significantly reduced efficiency to support SV40 *in vitro* DNA replication, while those made from sense transfected cells have fully capability of replication 51. Down-regulation of ANXA2 lead to reduction of the percentage of cells in the S phase 52. The results agree with that ANXA2 can promote cell proliferation by regulating DNA synthesis, replication and the cell cycles. Researchers also point out that ANXA2 activate both NF-κB and β-catenin signaling pathways thus causing cell proliferation *in vivo* 53.

### Roles of Annexin A2 in cell apoptosis

Inhibiting cell apoptosis are essential factor in tumor survival and proliferation. ANXA2 is significant ligand of C1q which directly binding to apoptotic cells and mediating phagocytes recognizing apoptosis cells 54, 55. P53 serves as a key tumor suppressor protein by preventing cellular transformation 56. The level of p53 impacts its target gene expression and regulates cellular functions such as cell cycle and apoptosis 57. ANXA2 influences p53 level by activating JNK/c-Jun signaling, thus suppress expression of p53 and its downstream genes, p21, GADD45 and BAX, which play roles in promoting apoptosis 58, 59. An experiment analysis the ANXA2 level of cells infected by p53 gene, it turns out that reduced expression was detected in all cell lines infected by Adv-p53 (a reconstructed adenovirus encoding wild type p53 gene) 60. In NSCLC cell lines, silencing ANXA2 led to up-regulation of p53 expression and inhibits cell proliferation 61. Knockdown of ANXA2 up-regulating the level of p53 and its downstream gene 62. In addition, it is also reported that knockdown of ANXA2 promoting the lung cancer cell apoptosis 63. Accumulating data suggest that ANXA2 participates in cellular apoptosis via regulating the expression level of p53.

### Roles of Annexin A2 in cell invasion and metastasis

Studies showed that down-regulating of ANXA2 inhibiting tumor cell invasion and metastasis, and overexpression of ANXA2, promote the invasion and metastasis capability of tumor cells 52, 64-69. Anti-ANXA2 antibody have been proved to serve as a suppressor in tumor invasion and metastases 59, 70-75. The ANXA2 heterotetramer specifically binding tPA on the surface of cell membrane and transform plasminogen (PLG) into plasmin that contribute to extracellular matrix (ECM) degradation and fibrin polymers lysis 76. Plasmin initiates neoangiogenesis and support tumor cell getting nutrition and oxygen 77, 78. Degradation of ECM and lysis of fibrin polymers provide space for migration. Membrane synthesis and cytoskeletal rearrangements, meanwhile, are key factors in process of migration. ANXA2 has the ability to bind polymerized and monomeric actin and maintain the plasticity of the dynamic membrane-associated actin cytoskeleton 79. In addition, epithelial-mesenchymal transition (EMT) is an essential process of metastasis in cancers. It provides stationary epithelial cells a chance to lose junctions with each other and be capable to migrate and invade 80-82. Downregulation of ANXA2 block cell proliferation and invasion accompanied by reduction of β-catenin and inhibition of EMT. While over expression of β-catenin reversed the negative effect on EMT 72. Transforming growth factor β (TGF-β) is an EMT inductor. TGF-β upregulates ANXA2 and activates internalization of both E-cadherin and ANXA2. ANXA2 overexpression promotes cell invasiveness through Src/ANXA2/STAT3 pathway. Silencing ANXA2 prevented TGF-β-induced invasion. Inhibition of Src/ANXA2/STAT3 pathway reversed the EMT process 83. Twist and Snail are recognized as key factors in EMT initiation. They decrease epithelial proteins (i.e., E-cadherin) and increase mesenchymal proteins (i.e., N-cadherin, fibronectin, β-catenin, and vimentin), providing a chance for cancer cells to escape from one organ to a different indirectly connected one 81. Study on level of twist and snail in NPC cells proved this mechanism 84. The level of twist and snail decreased in ANXA2-knockdown NPC cells, and those cells maintain the endothelial-like phenotype rather than a mesenchymal-like phenotype.

## Annexin A2 is an emerging biomarker for cancers

### Overexpression of Annexin A2 in cancer cell

**Figure 2:**
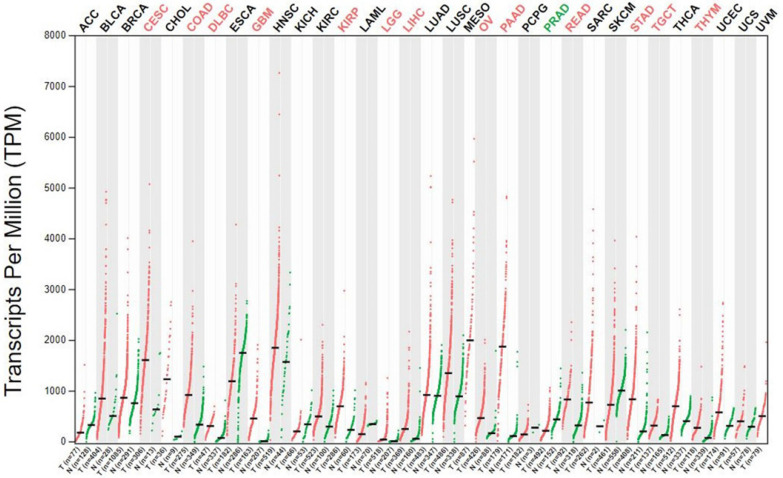
The expression of the ANXA2 gene in GEPIA database. ANXA2 is aberrantly expressed in a board range of cancer cells. The results consistent with the aforementioned studies.

Aberrant expression of ANXA2 is observed in extensive range of cancer cells. In breast cancer cell, expression of ANXA2 is detected in the metastatic MDA-MB231 cell but not in the nonmetastatic MCF-7 cell 85, 86. Overexpression of ANXA2 detected both in herceptin resistant and Her-2 negative breast cancer cells 87. In 105 cases of primary colorectal carcinoma tissues, ANXA2 is overexpressed in the cancer cell membrane of the carcinoma cells more than in tumor stroma fibrous tissue, the muscularis propria, the vessel wall, and the adjacent normal bowel wall. Similar results also showed in other colorectal cancers 88, 89. A clinical data collected 150 pairs of colorectal carcinoma tissue and the corresponding paracancerous tissue shows that ANXA2 is overexpressed in tumor cells and mainly located in the plasma membrane 90. A study based on immunohistochemistry provide evidence of ANXA2 presence in cancer samples, ANXA2 expression was increased in human tumor tissues. And ANXA2 levels were higher in stage IV and metastasis tumors compared with stage I-III. While E-cadherin, an epithelial marker, decreased in stage II-IV and increased in metastasis 83. Moreover, the expression of ANXA2 is positively correlated with histological type, tumor size, depth of invasion, and pathological tumor-node-metastasis stage. Higher level of ANXA2 is detected in human colorectal carcinoma cell lines including SW480, SW620, HCT116 and HT29 than normal colonic epithelial cell line NCM460 69. In 153 primary gastric carcinoma patients, about 30% are immunopositive for ANXA2 90. In another 436 gastric cancer cases, 133 gastric cancer tissue display upregulation of ANXA2. Additionally, ANXA2 expressed more strongly in the cell membrane than that in the cytoplasm of carcinoma cells 91. In terms of hepatocellular carcinoma (HCC), both the expression level and tyrosine phosphorylation of ANXA2 are upregulated in HCC compared to normal or cirrhosis tissue 92. Intense ANXA2 immunoreactivity is detected in lung adenocarcinoma, squamous cell carcinoma and non-small cell lung cancer (NSCLC) 59, 63, 93, 94. Additionally, increased expression of ANXA2 detected in acute promyelocytic leukemia (APL) 95-97, glioma 98, multiple myeloma (MM) 99, pancreatic cancer 100-102 and oral squamous cell carcinoma (OSCC) 103, 104. To further confirm expression of Annexin A2 gene in cancers, we performed bioinformatics analysis to detect Annexin A2 gene level in different cancer cells (*Figure 2*). ANXA2 overexpressed in colon adenocarcinoma (COAD), rectum adenocarcinoma (READ), liver hepatocellular carcinoma (LIHC), pancreatic adenocarcinoma (PAAD), stomach adenocarcinoma (STAD). The results are in accordance with above-mentioned studies. This analysis also indicates high expression of ANXA2 in cervical and endocervical cancers (CESC), lymphoid Neoplasm Diffuse Large B-cell Lymphoma (DLBC), glioblastoma multiforme (GBM), kidney renal papillary cell carcinoma (KIRP), brain lower grade glioma (LGG), ovarian serous cystadenocarcinoma (OV), testicular germ cell tumors (TGCT), thymoma (THYM).

### Prognostic and diagnostic significance of Annexin A2 in cancers

Detection of ANXA2 level is of interest due to its prognostic and diagnostic significance in cancer treatment. It is pointed out that ANXA2 overexpression in NSCLC 59, HCC 105, serous ovarian cancer 106, biopsies of epithelial ovarian cancer 107, urothelial carcinoma 108, breast cancer 109, 110 and nasopharyngeal carcinoma (NPC) 84 was associated with poor prognosis. A meta-analysis performed on 2321 patients with various cancers to confirm that high expression of ANXA2 was correlated with both overall survival (OS) (hazard ratio [HR] 1.56; p < 0.001) and disease-free survival (DFS) (HR 1.47; p < 0.001) 111. On the other hand, ANXA2 performs the function of a diagnostic factor for screening cancers. Increased ANXA2 serum level in peripheral blood has been evaluated in HCC 105, 112-114, gastric cancer 115 and OSCC 116. The data are described in *Table 1*. Besides excessive ANXA2 can be detected in sera of cancer patients, it is also reported that high ANXA2 expression is related to a high risk of metastases and recurrence 84, 117. It suggests that ANXA2 may represent a latent target of diagnosis and an emerging biomarker of prognosis in cancer therapy. Investigators have screened out a panel of probes, Tz6/10, and demonstrated those probes play the roles of cancer diagnosis and therapy by labeling and/or imaging ANXA2 from different cancer cell lines 118.

## Annexin A2 is a potential therapeutic target for cancers

It is known that ANXA2 expression is upregulated in board spectrum of cancer cells. ANXA2 perform important roles in tumor progression, including cell survival, proliferation, migration, invasion and metastatic. In addition, abnormal expression of ANXA2 is link to multidrug resistance in cancer treatment 41, 66, 93, 119-121. More attention has been focused on exploring therapies targeting ANXA2 in cancer treatment.

ANXA2 was identified a bleomycin binding site in the pulmonary fibrosis which result in resistance for bleomycin treatment 121. In the research, it is further proved that Glu139 (E139) of ANXA2 (ANXA2^E139A^) is required in bleomycin reduced pulmonary fibrosis. Mutating ANXA2^E139A^ in lung epithelial cells blocks the binding of bleomycin and ANXA2 and activates the transcription factor EB (TFEB) which regulates autophagy. TFEB-mediated autophagy substantially accelerates autophagic flux, leading to inhibition of epithelial cells apoptosis and proliferation, and ameliorates pulmonary fibrosis in bleomycin-treated mice. This founding makes ANXA2 a specific bleomycin target. Inhibiting ANXA2 may promote efficacy of bleomycin for cancer treatment.

Natural compounds are prospective directions in ANXA2-targeting therapy. Ginsenosides Rg5 and Rk1, having similar structure, have been found specifically binding to ANXA2. The interaction between those two ginsenosides and ANXA2 inhibit NF-κB activity and down-regulate inhibitor of apoptosis proteins, activating caspase and promoting apoptosis 122. Investigators purified plant lectin from chickpea (cicer arietinum agglutinin) and testified the anti-tumor efficacy. It turns out that the plant lectin can inhibit tumor cell proliferation, migration and promote apoptosis by block the binding of ANXA2 and galectin-3, causing suppression of EGFR-mediated signaling 123. Matrine, a plant alkaloid, purified from Chinese medical herb Sophora flavescens has been identified the anti-tumor activities by directly targeting ANXA2 124.

Investigators have designed and constructed a chemical modified DNA/RNA hybrid nanoparticle for ovarian cancer. This nanoparticle consists of a thiodeoxyribonucleic acid aptamer targeting ANXA2 and a highly thermodynamically stable three way junction (3WJ) core motif derived from pRNA of phi29 bacteriophage. The other arm of pRNA-3WJ is extended with GC rich sequences for doxorubicin loading. This construction maintains the property of targeting ANXA2 and the cell toxicity of doxorubicin. *In vivo* experiment, nanoparticles remained integrated construction and are selectively enriched in tumors while little accumulation in healthy organs 6 h post-injection 125. This result indicated that the novel cancer cell targeted drug delivery system may be a potential candidate to enhancing chemical drug efficiency in ovarian cancer treatment in an ANXA2-targeted manner. Another aptamer, ACE4G, is also demonstrated that it not only identifies ANXA2 on the membrane but realizes the enriched internalization into MCF-7 cell line 126.

It is reported that a cationic lipid-guided carrier with ANXA2 shRNA was designed to retarded tumor growth by silencing ANXA2, which have been proved a stable nanoparticle sustained targeting and localization in lung tumors nanoparticles both *in vitro* and *in vivo* 127.

Recently attentions have been focused on roles of microRNAs (miRNAs) in tumor regulations. MiR-206 has been testified directly targeting oncogenes KRAS and ANXA2 on tumor cell surface 128. Further study revealed that ANXA2 N-terminus, especially Tyr23, play crucial roles in maintaining the high malignancy of colonic adenocarcinoma and miR-206 act as a tumor suppressor in colonic adenocarcinoma 129. MiR-101 is also discovered being an ANXA2-targeted molecule and have capability of down-regulating expression of ANXA2 in drug-resistant gastric cancer 120.

Kim VM and his colleagues developed a Listeria-based, ANXA2-targeting cancer immunotherapy (Lm-ANXA2) and testing its efficacy for PDAC within two murine models. It turns out that PDAC model mouse treated with Lm-ANXA2 showed high survival rate, supporting the assumption that Lm-ANXA2 can serve as a targeted agent for PDAC treatment 130.

An anti-ANXA2 monoclonal antibody (mAb) 67 generated in lab showed significant suppression of cell growth of breast tumor *in vivo* 131. Simeon Cua and his colleagues generate a mAb, IgG1 2448, targeting a unique glycan epitope on ANXA2. It demonstrated good anti-tumor efficacy *in vivo* and indicated that 2448 can be a potential candidate of targeted therapy for ovarian and breast cancer 132, 133.

## A promising therapeutic strategy targeting Annexin A2

Therapeutic strategy targeting ANXA2 have showed significantly antitumor effect both *in vitro* and *in vivo* in preclinical study, barely any of those reach clinical trials, especially the anti-ANXA2. The probable reason is that surface epitopes on ANXA2 are challenging to recapitulate for generation purposes 132. In this review, we propose a prospective candidate for ANXA2-targeted therapeutic strategies, a targeting peptide selected via phage display technology showing highly selectivity and affinity to ANXA2.

There is a new peptide motif have been reported before 134, investigators discussed the LGRFYAASG peptide which is screened from internalizing phage peptide library in sarcoma cells. The peptide fused with the cell-penetrating peptide, pen motif, showed specific affinity and inhibitor effect in tumor cell. Notably, we get an ANXA2-targetting peptide motif - CBP12 (colorectal cancer binding peptide) in laboratory as well. We screened the phage display peptide libraries in human colorectal adenocarcinoma cell SW620 and human normal intestinal epithelial cell line HIEC (SW620 was used as the target cell and HIEC as the negative adsorption cell), collected the eluate and amplified by E. coli ER2738E infection. The CBP12 motif was specifically enriched after four rounds of selections. Consistently, we verified that phage display peptide CBP12 have specifically affinity to colorectal cancer cells. CBP12 can be a new targeting peptide and provide new directions in the early diagnosis and targeted therapies. Related articles will be published later.

Large number of literatures demonstrated the modification method of targeting peptide which inspire us the methods to improve the therapeutic effect of targeting peptide in tumor treatment. One way is to combine targeting peptide with classical killer peptide, KLA (KLAKLAKKLAKLAK), this kind of polypeptide showed favorable tumor cell toxicity and reduction of tumor volume *in vivo* with no apparent toxicities 135-137. KLA is a proptosis peptide, contributing to cell death via causing mitochondrial swelling and permeabilization and the release of cytochrome c, which disrupt mitochondrial membrane 138-142. While KLA barely permeate the eukaryotic plasma membrane separately because of its characteristic of cationic amphipathic 143. Accordingly, on the one hand, KLA requires the assistance of targeting peptides to transmembrane and play a role in programmed cell death. On the other hand, KLA shows no toxicity for normal cell as its low penetration in mammalian cells 144. Alternatively, the concept of antibody-drug conjugate (ADC) proposes different direction in cytotoxic payload link to the specific antibody 145-148. We can link the targeting peptide with the cytotoxic compounds which have been announced anti-tumor efficacy in clinical. Those compounds including auristatins, derivatives of dolastatin 10 149; maytansine, a potent microtubule inhibitor 150; calicheamicin, a DNA damaging agent causing DNA double strand breaks 151; duocarmycin and indolinobenzodiazepine pseudodimers, DNA damaging agents alkylating DNA 152, 153; and PBD dimers, a DNA damaging agents for cross linking DNA 154.

## Conclusions

It is known that ANXA2 overexpress in the surface of cancer cells and have been announced a biomarker of diagnosis and prognosis of cancers. ANXA2 serve as a potent target of therapeutic intervention and multiple therapeutic strategies targeting ANXA2 have been testified and showed favorable anti-tumor efficacy both *in vitro* and vivo. Here, we propose a promising targeting peptide with high affinity to ANXA2 selected from phage display technology. Based on the conception of polypeptide which comprised of targeting peptide and pro-apoptotic peptide and ADC which comprised of specific targeting antibody and toxicity compound, we pose a hypothesis that link the targeting peptide to a pro-apoptotic peptide or a mutual toxicity compound. The targeting peptide bind to ANXA2 on tumor cells and trigger the endocytosis, endosomes take the composition into cytoplasm and release the cytotoxic agent from the composition and exert cytotoxicity such as interrupting mitochondria, blocking cell division, damaging DNA.

## Figures and Tables

**Table 1:** ANXA2 serum level in peripheral blood

Cancer	Expression of ANXA2	References
HCC	ANXA2 in peripheral blood significantly increased in HCC patients (median, 69.6 ng/ml) compared to chronic liver disease patients (median, 16.8 ng/ml) and control group (median, 9.5 ng/ml) (p < 0.001)	Nevine El-Abd et al
HCC	Significantly increased ANXA2 is detected in the sera of HCC (median, 24.75 ng/µl) compared with that in the healthy (median, 16.69 ng/µl), benign tumor (median, 19.92 ng/µl), hepatitis (median, 6.48 ng/µl), and cirrhosis controls (median, 7.39 ng/µl)	Yulin Sun et al
HCC	Higher expression (t=10.32, P<0.001) was found in the HCC group (24.82±8.18 ng/mL) than those in the benign liver disease group (12.80±7.21 ng/mL)	Haijian Zhang et al
Early stage HCC	Serum ANXA2 level is 130 ng/ml compared with 15 ng/ml and 17 ng/ml in chronic liver disease patients and control group respectively	Mohamed K Shaker et al
Gastric cancer	ANXA2 levels in serum were significantly different between gastric cancer patients and control group (median, 211.0 vs. 120.5 μg/mL, respectively	Faruk Tas et al
OSCC	The ANXA2 level was significantly higher in OSCC patients (median, 27.1 ± 9.81 ng/mL) than in patients with benign disease and controls (median,15.9 ng/mL and 15.0 ng/mL, respectively)	Wei Zhang et al

† HCC, hepatocellular carcinoma; ‡ OSCC, oral squamous cell carcinoma.
